# Analysis of MCM Proteins’ Role as a Potential Target of Statins in Patients with Acute Type A Aortic Dissection through Bioinformatics

**DOI:** 10.3390/genes12030387

**Published:** 2021-03-09

**Authors:** Zheyong Liang, Yongjian Zhang, Qiang Chen, Junjun Hao, Haichen Wang, Yongxin Li, Yang Yan

**Affiliations:** Department of Cardiovascular Surgery, The First Affiliated Hospital of Xi’an Jiaotong University, Xi’an 710061, China; 008008@xjtufh.edu.cn (Y.Z.); 004027@xjtufh.edu.cn (Q.C.); 008288@xjtufh.edu.cn (J.H.); 000246@xjtufh.edu.cn (H.W.); liyongxin103@xjtu.edu.cn (Y.L.); yangyan3@xjtu.edu.cn (Y.Y.)

**Keywords:** aortic dissection, MCM proteins, statins, GEO, WGCNA

## Abstract

Acute aortic dissection is one of the most severe vascular diseases. The molecular mechanisms of aortic expansion and dissection are unclear. Clinical studies have found that statins play a protective role in aortic dissection development and therapy; however, the mechanism of statins’ effects on the aorta is unknown. The Gene Expression Omnibus (GEO) dataset GSE52093, GSE2450and GSE8686 were analyzed, and genes expressed differentially between aortic dissection samples and normal samples were determined using the Networkanalyst and iDEP tools. Weight gene correlation network analysis (WGCNA), functional annotation, pathway enrichment analysis, and the analysis of the regional variations of genomic features were then performed. We found that the minichromosome maintenance proteins (MCMs), a family of proteins targeted by statins, were upregulated in dissected aortic wall tissues and play a central role in cell-cycle and mitosis regulation in aortic dissection patients. Our results indicate a potential molecular target and mechanism for statins’ effects in patients with acute type A aortic dissection.

## 1. Introduction

Acute aortic dissection is a life-threatening disease. According to the Stanford system, aortic dissections can be classified as type A or B. Type A, which involves the ascending aorta, is the most threatening. The pathology of aortic dissection is the progressive separation of the aortic wall layers, which results in the formation of a false lumen. It can arise from a tear in the aortic intima, exposing the medial layer to the pulsatile blood flow. Blood flow in the false lumen can lead to an aortic rupture in the case of adventitial disruption or re-entry back into the true lumen through another intimal tear. Aortic rupture quickly leads to exsanguination and death. In the event of blood redirection into the true lumen, creating natural fenestration, the patient can present as relatively stable with adequate perfusion [1,2,3]. The pathogeny of aortic dissection includes two major parts. One is a pathogenic force on the aortic intima, mostly related to high pressure caused by uncontrolled hypertension. The other is low strength in the aortic wall, especially the intima and medial layer, which is usually associated with abnormal aortic development caused by congenital disease, injury, or other aortic lesions. Although aortic replacement can reduce the short-time mortality of the disease, complications can arise for the affected aorta, such as dilation, aneurysmal formation, and rupture, which remain problems in the chronic phase, and the long-term outcomes have not always been satisfactory [4,5,6]. The molecular mechanism of the tearing in the aortic intima or medial layer is still unclear, and the drugs that can stabilize the aortic wall before the dissection or after the surgery are limited.

Statins, 3-hydroxy3-methylglutaryl-coenzyme A reductase inhibitors, are therapeutic drugs widely used for hyperlipidemia. Increasing evidence suggests that they can inhibit aortic expansion and rupture [7,8], and they have been used to prevent the progression of aortic diseases [9,10,11]. It has been shown that the effect of statins on aortic dissection seems not to be based on the direct effect of lowering lipid levels, but their targets in aortic tissue are still unknown. Previous studies have shown that statins can inhibit minichromosome maintenance proteins (MCMs) and effectively influence cell survival in tumors [12,13]; however, MCMs’ role in aortic dissection is unknown.

Minichromosome maintenance proteins (MCMs) are highly conserved proteins involved in the initiation of eukaryotic genome replication. They can bind and translocate along DNA. They recruit other DNA-replication-related proteins and form the pre-replication complex (pre-RC), resulting in the unwinding of the DNA strand in the formation of replication forks [14,15,16]. The amount of MCMs in cells is limited. When cells are under replicative stress, the limited MCMs preclude cells from unlimited replication by causing DNA damage, which itself inhibits cell overgrowth via activating DNA damage checkpoint signaling, followed by DNA breaks [17]. In tumor cells, the MCMs are overexpressed and confer the potential for overgrowth [17]. Previous studies have found that statins can inhibit MCMs and tumor cell growth [12,13]; however, the expression and function of MCMs in the aorta have not been characterized.

## 2. Materials and Methods

### 2.1. Data Processing

In the Gene Expression Omnibus database (GEO, https://www.ncbi.nlm.nih.gov/geo/, accessed date: 16 November 2020) [18], we selected GSE52093, GSE2450 and GSE8686 for analysis. GSE52093 is based on the GPL10558 platform and includes 7 aortic dissection patients and 5 normal people’s aortic samples. GSE2450 is based on the GPL96 platform and includes samples of primary human umbilical vein endothelial cells (HUVECs) and immortalized HUVEC cell line EA.hy926 under different treatment. GSE8686 is based on the GPL2507 platform and includes samples of primary cultures of human microvascular endothelial cells (HMVEC), human pulmonary artery smooth muscle cells (PASMC), and normal human dermal fibroblasts (NHDF) under different treatment. Networkanalyst (https://www.networkanalyst.ca/, accessed date: 20 January 2021) [19,20,21] and iDEP (http://bioinformatics.sdstate.edu/idep/, accessed date: 24 May 2020) [22] were used to normalize and filter the gene expression profile in GSE52093.

### 2.2. Identification of Differentially Expressed Genes (DEGs)

The limma package [23] in R was used to identify differentially expressed genes (DEGs). The genes expressed differentially between aortic dissections and control were identified by using iDEP web tools. Fold changes > 2 and *p* < 0.05 adjusted by the false-discovery rate (FDR) were considered significant.

### 2.3. Weight Gene Correlation Network Analysis (WGCNA)

We extracted the expression profiles of genes commonly up- or downregulated in the aortic dissections/control to perform WGCNA [24] on GSE52093. The WGCNA R package in iDEP tools was used to construct co-expression networks (modules); the soft threshold was set to 5, the minimum module size was set to 20, and each module was assigned a unique color label. The Edge Threshold was set to 0.4, and the top 20 genes in the networks were shown. In addition, the iDEP web tools were used to perform functional enrichment analysis for these functional modules.

### 2.4. Analysis of Regional Variations of Genomic Features

The PREDA package [25] in iDEP was used to identify regional variations in the genomic data. The data were filtered with an FDR set to 0.1 and fold change set to 2. Enrichment analysis was then performed using the ShinyGO (http://ge-lab.org/go/, accessed date: 3 November 2019) web tools.

### 2.5. Immune-Cell Enrichment Analysis

The immune-cell enrichment analysis was conducted using xCell (https://xcell.ucsf.edu/, accessed date: 10 January 2020) [26], and the data were visualized using xCellView (https://comphealth.ucsf.edu/app/xcellview, accessed date: 10 January 2020). The Transform was set to Correlation, the Distance method was set to Euclidean, and the Clustering linkage was set to Mcquitty.

## 3. Results

### 3.1. Data Preprocessing and Normalization

**Figure 1:**
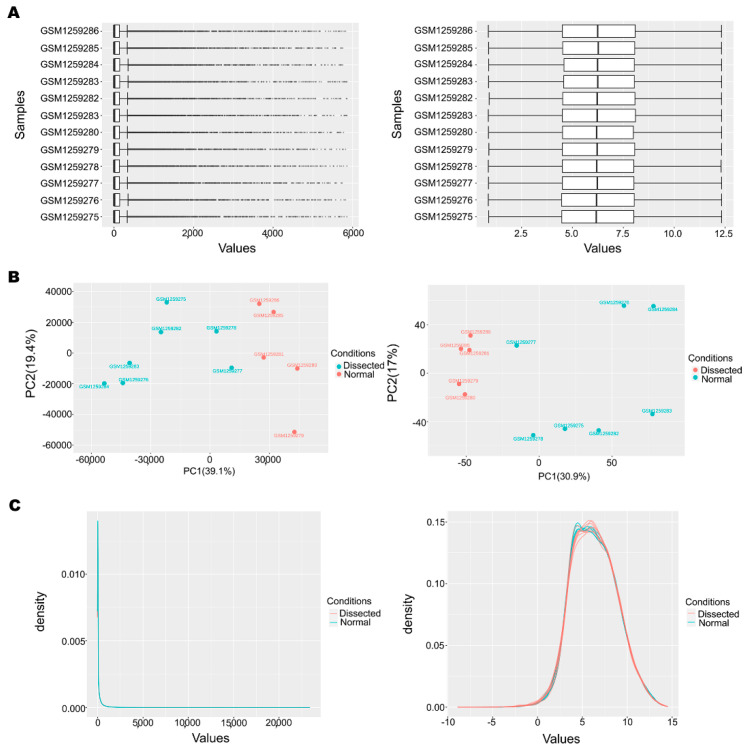
Data normalization. (**A**). The box plots of data before and after normalization. (**B**). The principal component analysis (PCA) plot of data before and after normalization. (**C**). The density plot of data before and after normalization.

**Figure 2:**
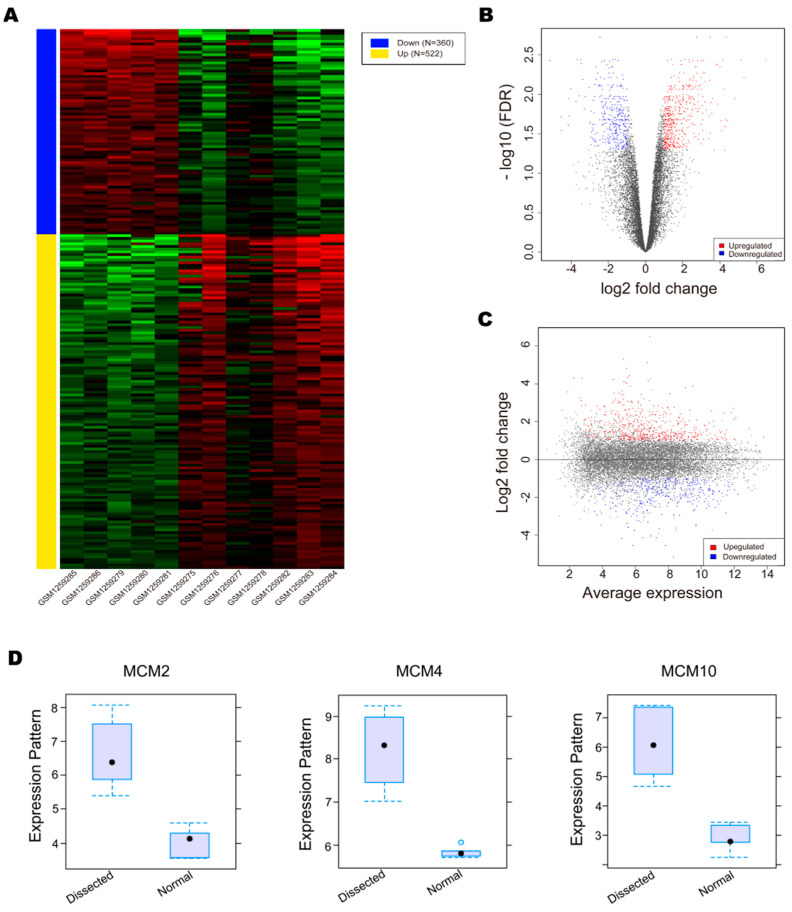
Identification of differentially expressed genes (DEGs) and the expression of minichromosome maintenance proteins (MCMs). (**A**). The heatmap of the DEGs. Blue: downregulated genes, *n* = 360; yellow: downregulated genes, *n* = 522. (**B**). The volcano plots of the DEGs. (**C**). The scatterplots of the DEGs. (**D**). The expression of MCM2, MCM4, and MCM10.

First, the gene expression matrix was normalized and processed. There were 24,106 genes in 12 samples; 11,872 genes passed the filter, and 8156 were converted to Ensembl gene IDs in the iDEP database. The remaining 3716 genes were kept in the data using the original IDs. The data distributions before and after normalization are presented by box plots in Figure 1A. Principal component analysis (PCA) of the matrix was performed before and after normalization and filtration (the plot is shown in Figure 1B). The relative distributions of the different groups before and after normalization are displayed in the density plot in Figure 2C. The results show that the clustering of the two groups of samples was more obvious after normalization, indicating that the sample source was reliable.

### 3.2. Identification of DEGs and the Expression of MCM Proteins

After data preprocessing, we used iDEP to identify DEGs. The results show 882 DEGs (with an FDR cutoff of 0.0500 and minimum fold change of 2), including 522 upregulated and 360 downregulated in the dissection groups compared with the normal groups (Figure 2A, Table S1). The DEGs in each of the comparisons were examined in detail using volcano plots and scatterplots (Figure 2B,C). Among the DEGs, we found that the MCM proteins were upregulated in the aortic dissection groups compared with the normal groups. The expression of MCM2, MCM4, MCM5, MCM6, and MCM10 was upregulated with an adjusted P-value less than 0.05, and the expression of MCM2, MCM4, and MCM10 was upregulated with logFC > 2 (Table 1). These results indicate that the expression of MCM proteins was significantly changed in the aortic dissection patients.

### 3.3. The Network of DEGs and the Correlation of the Genes with MCM Proteins

**Figure 3:**
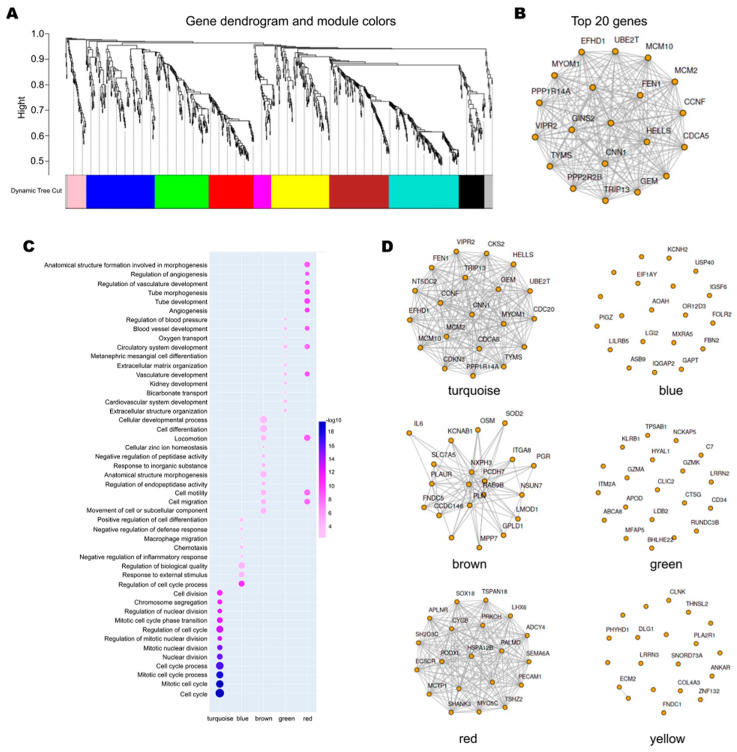
Weighted correlation network analysis. (**A**). Recognition module. Each module was given an individual color as an identifier, including ten different modules. (**B**). Top 20 genes of the entire network. (**C**). Biological process enrichment of genes in different modules. The significance of enrichment gradually increased from white to blue, and the dots’ sizes indicate the numbers of differentially expressed genes contained in the corresponding pathways. (**D**). Top 20 genes of different models.

To explore the co-expression network of the identified DEGs, weighted correlation network analysis (WGCNA) was performed using the iDEP web tools. A network of 973 genes was divided into nine modules (Figure 3A). MCM2 and MCM10 were found to be in the top 20 genes of the entire networks and in the top 20 of the turquoise module (Figure 3B,D). Then, Gene Ontology (GO) enrichment analyses were performed on these modules. Module function enrichment analysis showed that the turquoise module genes were significantly involved in biological processes related to the mitotic cell cycle and cell division. The blue module genes were significantly enriched in the biological processes of cell-cycle regulation and responses to external stimuli, whereas the brown module genes were significantly involved in the biological processes of cellular development and cell differentiation. The green module genes were significantly involved in biological processes related to circulatory system development and angiogenesis, whereas the red module genes were significantly involved in cell motility, cell migration, tube development, and morphogenesis (Figure 3C).

### 3.4. The Regional Variations of Genomic Features of Aortic Dissection

**Figure 4:**
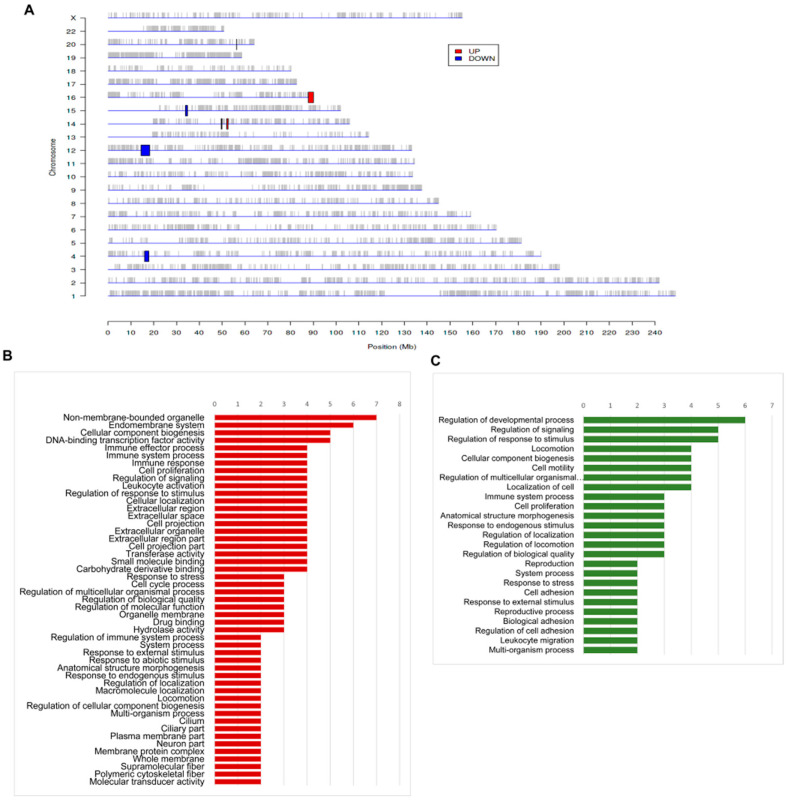
The regional variations of genomic features. (**A**). Amplified (red) and deleted (blue) regions identified by PREDA. (**B**). The biological process classification of genes on chr16q24 (amplified region). (**C**). The biological process classification of genes on chr12p12 (deleted region).

As MCMs play an important role in forming the pre-replication complex that regulates eukaryotic genome replication, we used the PREDA package in iDEP to identify genomic regions significantly enriched with up- or downregulated genes. We found that amplified regions were found in chr14q21, chr14q22, and chr16q24, while deleted regions were found in chr4p15, chr12p12, and chr15q14 (Figure 4, Table 2). Furthermore, we investigated the upregulated genes on chr16q24 and the downregulated genes on chr12p12 with ShinyGO v0.61 and found that genes associated with cell proliferation and the endomembrane system were upregulated. By contrast, genes associated with cell motility and cell adhesion were downregulated.

### 3.5. Statins Inhibit MCM Proteins Expression in Vascular Cell

**Figure 5:**
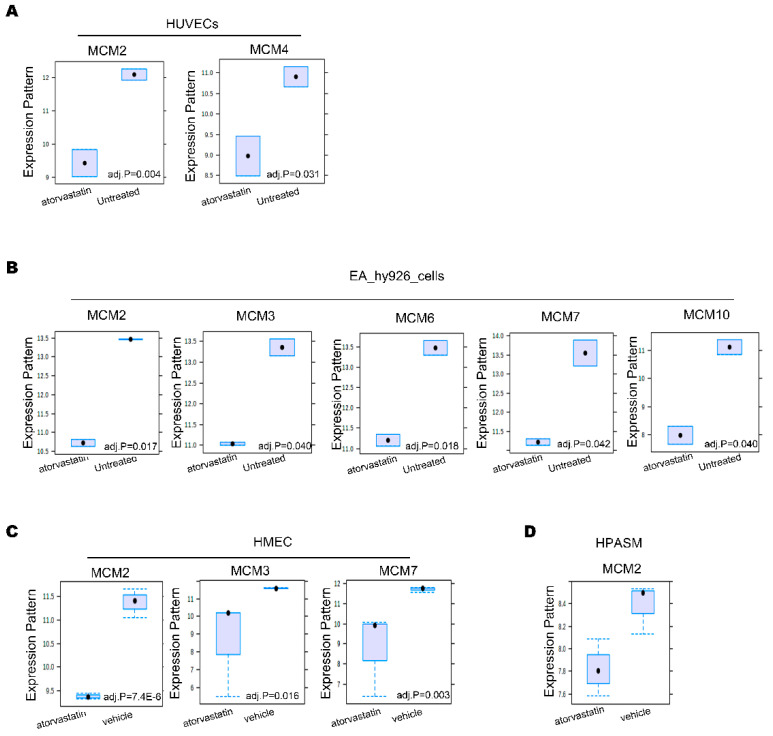
The effect of atorvastatin on MCMs in vascular cells. (**A**). The expression of MCM2 and MCM4 in primary human umbilical vein endothelial cells (HUVECs). (**B**). The expression of MCM2,3,6,7,10 in immortalized HUVEC cell line EA.hy926. (**C**). The expression of MCM2, MCM3 and MCM7 in primary cultures of human microvascular endothelial cells (HMVEC). (**D**). The expression of MCM2 in primary cultures of human pulmonary artery smooth muscle cells (PASMC).

Previous studies found that statins can inhibit the expression of MCM proteins. To confirm statins’ effects on MCM proteins in cardiovascular system, we further analyzed two different datasets GSE2450 and GSE8686. GSE2450 includes samples of primary human umbilical vein endothelial cells (HUVECs) and immortalized HUVEC cell line EA.hy926 treated by atorvastatin. GSE8686 includes samples of primary cultures of human microvascular endothelial cells (HMVEC), human pulmonary artery smooth muscle cells (PASMC), and normal human dermal fibroblasts (NHDF) treated by atorvastatin. Both data were normalized and processed. Then, we used Networkanalyst to identify DEGs between atorvastatin treatment and control. we found that, in HUEVCs the expression of MCM2 and MCM4 was significantly downregulated under atorvastatin treatment (Figure 5A), and in EA.hy926 cells the expression of MCM2,3,6,7,10 in atorvastatin treatment groups were much lower than that in control groups (Figure 5B). Additionally, in HMECs the expression of MCM2, MCM3 and MCM7 was significantly downregulated under atorvastatin treatment (Figure 5C). In HPASM cells the expression of MCM2 in atorvastatin treatment groups was also lower than that in control groups (Figure 5D). These results indicated that in endothelial cells and smooth muscle cells, statins could also inhibit the expression of MCMs.

## 4. Discussion

Acute aortic dissection is a fatal pathology that occurs in the aortic wall [27]. Its incidence is much higher in developing than developed countries. Although more and more patients are surviving through receiving surgical repair, many still develop aortic aneurysms or dissections after surgery. Clinical studies have found that statins inhibit aortic arch dilatation and could prevent aortic dissection [28]; however, how statins affect aortic dissection development remains unclear. Previous studies have found that statins can suppress MCM proteins and, thereby, inhibit tumor cell growth [12,13,17]. In this study, we analyzed GEO data for acute aortic dissection and found that MCM proteins were upregulated in aortic dissections. We also found that MCM2, MCM4, and MCM10 were strongly correlated with aortic dissections and mostly participated in processes related to the regulation of the cell cycle and division. Furthermore, we investigated the differences in genomic features between aortic dissections and normal aortas. We found that genes in the amplified region were associated with cell proliferation and the endomembrane system, whereas genes in the deletion region were associated with cell motility and adhesion. These results indicate that MCM proteins are important in aortic dissection development.

**Figure 6:**
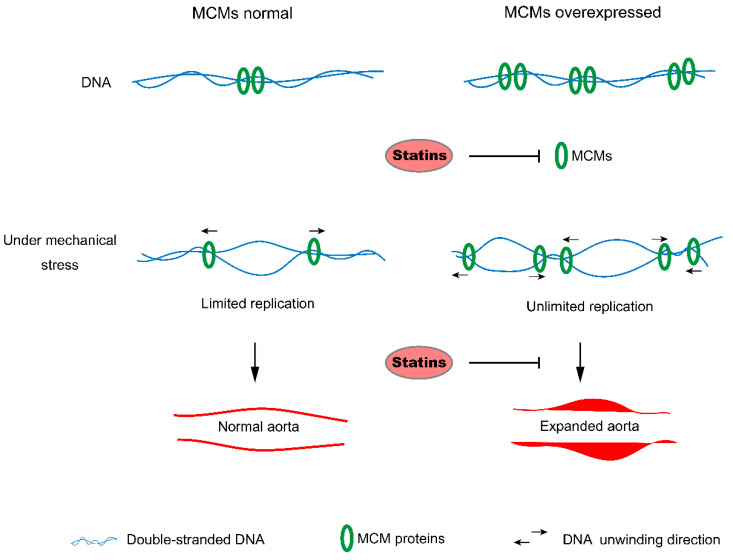
The model of MCM proteins’ role in the progression of aortic expansion. Under replication stress caused by mechanical stress such as hypertension, the limited MCM proteins in the normal aorta can slow down the proliferation of endothelial or smooth muscle cells through active DNA damage checkpoints. By contrast, upregulated MCM proteins may cause overproliferation of endothelial and smooth muscle cells and cause aortic expansion. Statins may prevent aortic expansion by targeting MCM proteins and inhibit the overreplication of cells.

Our WGCNA network analysis showed that the top DEGs were mostly enriched in processes related to cell-cycle control. Tao Jiang et al. also found that cell-cycle and Extracellular matrix (ECM)-receptor pathways might play important roles in type A acute aortic dissection (AAAD) [29]; this is consistent with our results. The MCMs are a group of proteins that play a key role in DNA replication, the most important step of cell division. MCM2–7 form a hexamer that can bind to DNA and form a pre-replication complex. It translocates along the DNA, resulting in the unwinding of the DNA strand, and then forms a replication fork [16] at a replication origin during the early G1 phase [30,31,32,33], and it is responsible for the correct licensing of DNA. MCM10 can interact with MCM2, MCM6, and the origin recognition protein ORC2 to recognize the origin of the DNA strand. Ibarra et al. demonstrated that the knockdown of any of the MCM complex subunits (MCM2–7) leads to the dysfunction of the whole complex and reduces the backup capacity for DNA licensing, which then leads to the abnormal replication of DNA during the S phase and activates the DNA damage response (DDR), stopping the cell cycle [17]. Our results show that MCM proteins were upregulated in the aortic dissection tissues. This indicates that the patients’ aortic endothelial cells or smooth muscle cells might have had a larger backup capacity for DNA licensing than normal people’s cells. Under replication stress, limited MCM proteins in normal aortas could slow down the proliferation of endothelial or smooth muscle cells through active DNA damage checkpoints. Upregulated MCM proteins may cause the overproliferation of endothelial and smooth muscle cells and cause aortic expansion (Figure 6).

Previous studies have shown that statins can inhibit the MCM complex in tumor cells [12,13]. Statins can inhibit cell growth and induce apoptosis by reducing chromosomal instability both in vitro and in vivo. In our study, we found that MCM proteins were significantly upregulated and play an important role in aortic dissections. The overexpressed MCMs may lead to unlimited DNA replication and then induce chromosome instability in endothelial and smooth muscle cells under stress conditions. It should be mentioned that, the change in the expression of the most statin-sensitive according to the literature MCM7 was found here was statistically insignificant. It has been found that the expression of MCM complex proteins is highly correlated. The inhibition of one single MCM complex protein will lead to the downregulation of other members [17]. Furthermore, our analysis data shown that, except MCM7, other MCM proteins such as MCM2, MCM6 and MCM10 were also downregulated under statin treatment. Therefore, we believe that the MCM proteins function together as a complex and could be destroyed by statins. Therefore, we predicted that MCM proteins might act as a target of statins in the inhibition of aortic dissection. Statins may inhibit the overexpressed MCMs and then normalize the genome stability. In fact, our analysis of the regional variations of genomic features showed that the genes located in the amplification or deletion regions participated in the aortic expansion process. These results indicate that chromosomal instability might participate in aortic dissection development, and statins may help to reduce this instability. Louis H. Stein et al. showed that statins have a protective effect on thoracic aneurysms. The adverse events (i.e., death, dissection, or rupture) and surgery rates for patients receiving statin therapy were lower than those for patients not receiving statin therapy [34,35]. Junichi Tazaki et al. found that the use of statins was associated with a lower risk for the primary outcome measure in patients with aortic dissection [36]. Recently, Naoki Masaki et al. also found that pitavastatin treatment, in addition to standard antihypertensive therapy, may have a suppressive effect on aortic arch dilatation in patients with acute type B aortic dissection [37]; however, the mechanism of statins’ effects on aortic dissection is unclear. Some studies have shown that statins can prevent cardiovascular diseases by protecting endothelia [34]. Yuki Izawa-Ishizawa et al. showed that pitavastatin can help to prevent endothelial dysfunction and aortic dissection in mice [38]. It may function through regulating inflammation. In our study, the enrichment analysis showed that MCM10, MCM2, and MCM4 also participated in the process of immune regulation (Table S2). We also analyzed the data by immune-cell-type enrichment and noted the immune-cell types associated with aortic dissection (Table S3); however, whether statins could affect those genes needs further study.

## 5. Conclusions

In summary, our studies showed that MCM2, MCM4, and MCM10 play an important role in aortic dissection and the expression of MCMs was inhibited by atorvastatin treatment. It indicated that MCMs may serve as potential target of statins in the prevention of aortic expansion.

## Figures and Tables

**Table 1:** MCM expression in aortic dissections vs. normal controls.

Gene	logFC	adj-P-Val
MCM2	2.62753	0.00817
MCM3	0.66044	0.08468
MCM4	2.37032	0.00656
MCM5	1.52354	0.03502
MCM6	0.86740	0.01988
MCM7	0.78220	0.06388
MCM10	3.21558	0.00684

**Table 2:** The regional variations of genomic features of aortic dissection.

Regulation	Chr.	Start	End	Size	Band	Genes
Up	14	49622140	49895068	0.273	q21	POLE2, KLHDC1, ARF6
Up	14	52037015	52595274	0.558	q22	PTGER2, TXNDC16, GPR137C
Up	16	87849765	90074319	2.225	q24	ZNF469, ZFPM1, CYBA, MVD, RNF166, CTU2, APRT, GALNS, TRAPPC2L, ZNF778, SNORD68; CHMP1A, CDK10, SPATA2L, ZNF276, MC1R, TUBB3, CENPBD1, DBNDD1, GAS8, PRDM7
Down	4	16023727	17805706	1.782	p15	TAPT1, LDB2, QDPR, LAP3, MED28, DCAF16
Down	12	14434283	18200488	3.766	p12	C12orf60, ART4, MGP, ERP27, ARHGDIB, RERG, PTPRO, EPS8, STRAP, MGST1, LMO3, RERGL
Down	15	33952715	34792889	0.84	q14	PGBD4, KATNBL1, SLC12A6, NOP10, LPCAT4, GOLGA8A, GOLGA8B, ACTC1

## Data Availability

The data supporting the reported results can be found in the article.

## Supplementary Materials

The following are available online at https://www.mdpi.com/2073-4425/12/3/387/s1. Table S1: The genes expressed differentially between aortic dissections and control, Table S2: The process of immune regulation, Table S3: The immune-cell types associated with aortic dissection.

## References

[1] Vilacosta I., Aragoncillo P., Canadas V., Roman J.A.S., Ferreiros J., Rodriguez E. (2010). Acute aortic syndrome: A new look at an old conundrum. Postgrad. Med. J..

[2] White A., Broder J., Mando-Vandrick J., Wendell J., Crowe J. (2013). Acute Aortic Emergencies—Part 2 Aortic Dissections. Adv. Emerg. Nurs. J..

[3] Gawinecka J., Schönrath F., Von Eckardstein A. (2017). Acute aortic dissection: Pathogenesis, risk factors and diagnosis. Swiss Med. Wkly..

[4] Suzuki T., Isselbacher E.M., Nienaber C.A., Pyeritz R.E., Eagle K.A., Tsai T.T., Cooper J.V., Januzzi J.L., Braverman A.C., Montgomery D.G. (2012). Type-Selective Benefits of Medications in Treatment of Acute Aortic Dissection (from the International Registry of Acute Aortic Dissection [IRAD]). Am. J. Cardiol..

[5] Durham C.A., Cambria R.P., Wang L.J., Ergul E.A., Aranson N.J., Patel V.I., Conrad M.F. (2015). The natural history of medically managed acute type B aortic dissection. J. Vasc. Surg..

[6] Akutsu K., Nejima J., Kiuchi K., Sasaki K., Ochi M., Tanaka K., Takano T. (2004). Effects of the patent false lumen on the long-term outcome of type B acute aortic dissection. Eur. J. Cardio-Thoracic Surg..

[7] Kalyanasundaram A., Elmore J.R., Manazer J.R., Golden A., Franklin D.P., Galt S.W., Zakhary E.M., Carey D.J. (2006). Simvastatin suppresses experimental aortic aneurysm expansion. J. Vasc. Surg..

[8] Steinmetz E.F., Buckley C., Shames M.L., Ennis T.L., Vanvickle-Chavez S.J., Mao D., Goeddel L.A., Hawkins C.J., Thompson R.W. (2005). Treatment with Simvastatin Suppresses the Development of Experimental Abdominal Aortic Aneurysms in Normal and Hypercholesterolemic Mice. Ann. Surg..

[9] Takagi H., Matsui M., Umemoto T. (2010). A meta-analysis of clinical studies of statins for prevention of abdominal aortic aneurysm expansion. J. Vasc. Surg..

[10] Raux M., Cochennec F., Becquemin J.-P. (2012). Statin therapy is associated with aneurysm sac regression after endovascular aortic repair. J. Vasc. Surg..

[11] Sukhija R., Aronow W.S., Sandhu R., Kakar P., Babu S. (2006). Mortality and Size of Abdominal Aortic Aneurysm at Long-Term Follow-Up of Patients Not Treated Surgically and Treated with and Without Statins. Am. J. Cardiol..

[12] Liang Z., Li W., Liu J., Li J., He F., Jiang Y., Yang L., Li P., Wang B., Wang Y. (2017). Simvastatin suppresses the DNA replication licensing factor MCM7 and inhibits the growth of tamoxifen-resistant breast cancer cells. Sci. Rep..

[13] Li J., Liu J., Liang Z., He F., Yang L., Li P., Jiang Y., Wang B., Zhou C., Wang Y. (2017). Simvastatin and Atorvastatin inhibit DNA replication licensing factor MCM7 and effectively suppress RB-deficient tumors growth. Cell Death Dis..

[14] Tye B.K. (1999). MCM Proteins in DNA Replication. Annu. Rev. Biochem..

[15] Maiorano D., Lutzmann M., Méchali M. (2006). MCM proteins and DNA replication. Curr. Opin. Cell Biol..

[16] Enemark E.J., Joshua-Tor L. (2008). On helicases and other motor proteins. Curr. Opin. Struct. Biol..

[17] Ibarra A., Schwob E., Méndez J. (2008). Excess MCM proteins protect human cells from replicative stress by licensing backup origins of replication. Proc. Natl. Acad. Sci. USA.

[18] Barrett T., Wilhite S.E., Ledoux P., Evangelista C., Kim I.F., Tomashevsky M., Marshall K.A., Phillippy K.H., Sherman P.M., Holko M. (2013). NCBI GEO: Archive for functional genomics data sets—Update. Nucleic Acids Res..

[19] Zhou G., Soufan O., Ewald J., Hancock R.E.W., Basu N., Xia J. (2019). NetworkAnalyst 3.0: A visual analytics platform for comprehensive gene expression profiling and meta-analysis. Nucleic Acids Res..

[20] Xia J., Benner M.J., Hancock R.E.W. (2014). NetworkAnalyst—Integrative approaches for protein–protein interaction network analysis and visual exploration. Nucleic Acids Res..

[21] Xia J., E Gill E., Hancock R.E.W. (2015). NetworkAnalyst for statistical, visual and network-based meta-analysis of gene expression data. Nat. Protoc..

[22] Ge S.X., Son E.W., Yao R. (2018). iDEP: An integrated web application for differential expression and pathway analysis of RNA-Seq data. BMC Bioinform..

[23] Ritchie M.E., Phipson B., Wu D., Hu Y., Law C.W., Shi W., Smyth G.K. (2015). limma powers differential expression analyses for RNA-sequencing and microarray studies. Nucleic Acids Res..

[24] Langfelder P., Horvath S. (2008). WGCNA: An R package for weighted correlation network analysis. BMC Bioinform..

[25] Ferrari F., Solari A., Battaglia C., Bicciato S. (2011). PREDA: An R-package to identify regional variations in genomic data. Bioinformatics.

[26] Aran D., Hu Z., Butte A.J. (2017). xCell: Digitally portraying the tissue cellular heterogeneity landscape. Genome Biol..

[27] Nienaber C.A., Clough R.E., Sakalihasan N., Suzuki T., Gibbs R., Mussa F., Jenkins M.P., Thompson M.M., Evangelista A., Yeh J.S.M. (2016). Aortic dissection. Nat. Rev. Dis. Prim..

[28] Members T.F., Erbel R., Aboyans V., Boileau C., Bossone E., Di Bartolomeo R., Eggebrecht H., Evangelista A., Falk V., Frank H. (2014). 2014 ESC Guidelines on the diagnosis and treatment of aortic diseases. Eur. Hear. J..

[29] Jiang T., Si L. (2019). Identification of the molecular mechanisms associated with acute type A aortic dissection through bioinformatics methods. Braz. J. Med Biol. Res..

[30] Todorov I.T., Attaran A., E Kearsey S. (1995). BM28, a human member of the MCM2-3-5 family, is displaced from chromatin during DNA replication. J. Cell Biol..

[31] Young M.R., Tye B.K. (1997). Mcm2 and Mcm3 are constitutive nuclear proteins that exhibit distinct isoforms and bind chromatin during specific cell cycle stages of Saccharomyces cerevisiae. Mol. Biol. Cell.

[32] Krude T., Musahl C., A Laskey R., Knippers R. (1996). Human replication proteins hCdc21, hCdc46 and P1Mcm3 bind chromatin uniformly before S-phase and are displaced locally during DNA replication. J. Cell Sci..

[33] Aparicio O.M., Weinstein D.M., Bell S.P. (1997). Components and Dynamics of DNA Replication Complexes in S. cerevisiae: Redistribution of MCM Proteins and Cdc45p during S Phase. Cell.

[34] Torrens C., Kelsall C.J., Hopkins L.A., Anthony F.W., Curzen N.P., Hanson M.A. (2009). Atorvastatin Restores Endothelial Function in Offspring of Protein-Restricted Rats in a Cholesterol-Independent Manner. Hypertension.

[35] Stein L.H., Berger J., Tranquilli M., Elefteraides J.A. (2013). Effect of Statin Drugs on Thoracic Aortic Aneurysms. Am. J. Cardiol..

[36] Tazaki J., Morimoto T., Sakata R., Okabayashi H., Yamazaki F., Nishiwaki N., Mitsudo K., Kimura T. (2014). Impact of statin therapy on patients with coronary heart disease and aortic aneurysm or dissection. J. Vasc. Surg..

[37] Masaki N., Kumagai K., Sasaki K., Matsuo S., Motoyoshi N., Adachi O., Akiyama M., Kawamoto S., Tabayashi K., For the STANP trial investigators (2018). Suppressive effect of pitavastatin on aortic arch dilatation in acute stanford type B aortic dissection: Analysis of STANP trial. Gen. Thorac. Cardiovasc. Surg..

[38] Kohno M., Shinomiya K., Abe S., Noma T., Kondo I., Oshita A., Takeuchi H., Takagi Y., Yukiiri K., Mizushige K. (2002). Inhibition of Migration and Proliferation of Rat Vascular Smooth Muscle Cells by a New HMG-CoA Reductase Inhibitor, Pitavastatin. Hypertens. Res..

